# Synergistic effects of quercetin and regular exercise on the recovery of spatial memory and reduction of parameters of oxidative stress in animal model of Alzheimer's disease

**DOI:** 10.17179/excli2019-2082

**Published:** 2020-05-08

**Authors:** Amin Molaei, Homeira Hatami, Gholamreza Dehghan, Reihaneh Sadeghian, Nazli Khajehnasiri

**Affiliations:** 1 Department of Animal Biology, Faculty of Natural Sciences, University of Tabriz, Tabriz, Iran; 2 Medical Plants Research Center, Basic Health Sciences Institute, Shahrekord University of Medical Sciences, Shahrekord, Iran; 3 Department of Pharmacy, Faculty of Medicine, Mashhad University of Medical Sciences, Mashhad, Iran; 4 Department of Biological Sciences, Faculty of Basic Sciences, Higher Education Institute of Rab-Rashid, Tabriz, Iran

**Keywords:** Alzheimer Disease, streptozotocin, quercetin, exercise, oxidative stress, hippocampus

## Abstract

It has widely been reported that the brain in Alzheimer's disease (AD) is affected by increased oxidative stress, and this may have a role in the pathogenesis of this disorder. Quercetin, a polyphenol extensively found in nature, has recently been considered. Also, physical activities have a paradoxical effect on brain function in older adults. Therefore, this study aimed at investigating the synergic effects of quercetin (as chemical treatment) and exercise (as physical treatment) on AD-induced learning and memory impairment. Fifty-six adult male Wistar rats were randomly assigned into one of the following eight groups (n=7): The Control, Sham (saline), AD (intracerebroventricular administration of streptozotocin (STZ)), AD+80 mg/kg Quercetin (STZ+Q80), Quercetin vehicle (1 % Ethanol)+STZ, Exercise pretreatment (EX)+STZ, Off the treadmill+STZ, and EX+Q80+STZ. Quercetin administration was done intraperitoneally for 21 days after STZ injection. The rats ran on the treadmill for one hour a day for 60 days at a speed of 20-22 m/min. After the treatment, the spatial memory and levels of oxidative stress parameters were evaluated. The results showed that STZ caused spatial memory impairment and increased oxidative stress in the hippocampus. Exercise pretreatment or Quercetin injection improved the spatial memory impairment and oxidative stress caused by STZ injection. However, the combination of quercetin and exercise pretreatment was more effective. It can be concluded that the combined exercise pretreatment and Quercetin injection affected the antioxidant defense system and improved STZ-induced memory impairment.

## Introduction

The brain biochemical integrity is essential for the function of the central nervous system (CNS). Oxidative stress is one of the parameters contributed to cerebral biochemical impairment. The brain, because of the high oxygen consumption and lipid-rich content, is highly susceptible to oxidative stress. Consequently, oxidative stress can alter normal CNS functions (Salim, 2017[45]). 

The role of oxidative stress in the pathophysiology of neurodegenerative disorders, such as Alzheimer's disease (AD) has been investigated (Markesbery, 1999[35]). In AD, oxidation of the brain’s lipids, carbohydrates, proteins, and DNA is increased (Huang et al., 2016[24]), which increases the generation and accumulation of amyloid-beta (Aβ) peptides (Cheignon et al., 2018[12]).

The loss of spatial memory, as an early clinical symptom of AD, is due to synaptic dysfunction rather than neuronal death. In AD patients, the impairments of spatial memory are associated with a decline in the excitatory pathway (Zhu et al., 2017[60]). Besides, in these patients, cerebrospinal fluid proteins are accompanied by an early increase in Aβ42, following a decrease in t-tau and p-tau (Antonell et al., 2011[3]) and Aβ-degrading enzymes in the brain (Reddy et al., 2010[41]). CSF biomarkers have also provided good diagnostic accuracy and prediction of progress for mild cognitive impairment (MCI), a transition period between healthy aging and dementia (Ferreira et al., 2014[16]). 

Inconsistent results have been reported regarding the possible effects of exercise on aging (Basso and Suzuki, 2017[6]). There is a bulk of literature showing the positive association between physical activity and brain functions in MCI and AD at least in humans has been approved (it should be noted that there is no effective pharmacological treatment for MCI). It has been shown that six months of high-intensity aerobic exercise in MCI patient increased neuropsychological test function and reduced the risk of dementia (Smith et al., 2013[53]). Other investigations have stated that at least 150 min of moderate aerobic physical activity per week in combination with resistance training is necessary to maintain the brain’s health in MCI patients aged 65–89 years and led to improving cognitive status in MCI patients (Lautenschlager et al., 2019[31]). Also, moderate exercise during midlife was correlated with a 39 % lower risk of having MCI in later life. Late-life moderate exercise was associated with a 32 % lower risk for MCI (Geda et al., 2010[19]). The neural mechanisms responsible for the effect of physical activity on cognition are neurogenesis, angiogenesis, synaptogenesis, and the effect of neurotrophins (Joubert and Chainay, 2018[25]). 

Physical activity also can have positive or negative (Finaud et al., 2006[17]) effects or even no effect (Hadzovic-Dzuvo et al., 2014[23]) on oxidative stress, depending on its load, specificity, type, and even gender, race, and aging (Finaud et al., 2006[17]).

Quercetin is a flavonoid, which is highly distributed in fruits and vegetables and has been found with antioxidant properties (Ansari et al., 2009[2]). However, it has been found that under a balanced state, quercetin exerted antioxidant effects on impaired cognition (Xia et al., 2015[59]). Several pathways have been proposed, through which quercetin may influence cognitive performance. As an example, *in vitro* studies have declared that quercetin is a powerful antioxidant, and may protect neuronal cells from neurotoxicity due to oxidative stress. Also, quercetin is an adenosine antagonist that may reduce age-related cognitive impairments of aging (Broman-Fulks et al., 2012[9]).

Different results have been reported regarding the used dose of quercetin. For example, it has been shown that quercetin at lower doses has ameliorated the diabetes-induced changes in oxidative stress directly and indirectly by suppressing risk factors for dementia, such as hyperglycemia (Mahesh and Menon, 2004[34]). Another study has indicated that higher concentrations of quercetin could completely reverse cognitive decline induced by a high-fat diet. On the other hand, all doses of quercetin can attenuate oxidative stress in the neurodegenerative diseases affecting the hippocampus (Xia et al., 2015[59]).

In previous studies, antioxidants and exercise alone and with different protocols have been used to prevent and treat AD and the obtained results have been relatively satisfactory; however, the synergic effect of these factors on AD needs further studies. Therefore, the purpose of the present study was to investigate the presence or absence of synergistic effects between one of the most commonly used antioxidants called quercetin and regular exercise on spatial memory improvement in animal models of AD.

## Material and Methods

### Ethics statement

Animal care, treatment, and surgical procedures were approved by the scientific and ethics committees of the Tabriz University of Medical Sciences and performed based on the Guide for Care and Use of Laboratory Animals published by the National Institute of Health, United States (NIH Publication No. 85-23, revised 1985). All efforts have been made to minimize suffering. Operations that could create pain and distress were performed in the absence of other animals. Rats were anesthetized by intraperitoneal (i.p.) injection of a mixture of xylazine (10 mg/kg) and ketamine (100 mg/kg) before sacrifice and at the end of experiments, they were killed by inhalation of CO_2_.

### Animals

Fifty-six adult male Wistar rats (230 ± 20 g, age: 3-4 months) were obtained from the animal house of the Institute Pastor of Tehran, Iran. Animals were maintained in a 12:12-h light-dark cycle (light on between 7:00 a.m. and 7:00 p.m.) and food and water were available ad libitum. 

The rats were chosen randomly. At first, eight cages were selected and then the treatment procedure that was written on pieces of paper was randomly placed inside each cage. Next, the rats were assigned one by one to different cages. Finally, animals with different treatment groups were housed in identical shelves in one room.

The animals were adopted and taken to their new environment one week before the beginning of the test. The rats were randomly allocated into the following eight groups (n = 7 per group): (1) The control group (without any intervention); (2) Sham group receiving saline (5 μL; intracerebroventricular (i.c.v.) injection) during operation followed by receiving saline (5 μL; i.c.v.; 21 days); (3) AD +saline group, receiving STZ (3 mg/kg, 5 µL, i.c.v.; Sigma-Aldrich) during operation followed by receiving saline; i.p; (4) STZ+quercetin group (80 mg/kg, i.p; Sigma-Aldrich), receiving STZ through i.c.v. injection during operation and then receiving quercetin for seven days after STZ injection until the 21^st^ day; (5) Quercetin vehicle (1 % Ethanol)+ STZ group, receiving STZ through i.c.v. injection during operation followed by ethanol treatment through i.p. injection, seven days after STZ injection until the 21^st^ day; (6) Exercise pretreatment (EX)+STZ group that started running on a treadmill (20-22 m/min) 60 days before the induction of AD for one hour; (7) Off the treadmill+STZ group that placed on a non-moving treadmill for 60 days followed by i.c.v. injection of STZ; and (8) EX+Q80+STZ group (the rats with AD that received pretreatment exercise, as well as quercetin).

### Surgical techniques for intracerebro-ventricular injection

To this objective, the sham and experimental rats were anesthetized intraperitoneally with ketamine (100 mg/kg) plus xylazine (10 mg/kg). The animals were placed in a stereotaxic device (Stoelting, USA) and their scalps were removed. Thereupon, lateral ventricles were drilled according to the Paxinos and Watson atlas (Shahidi et al., 2018[49]). Then, a guide cannula was placed inside two lateral ventricles and its connected cement was located on the scratched skull that was made stable by stitching. The microinjection of the drug or vehicle injection was performed using a 30-gauge injector cannula and a Hamilton syringe connected to the injector cannula through polyethylene micro-tubing (Shahidi et al., 2018[48]). To induce AD, STZ powder (3 mg/kg) was dissolved in normal saline and injected at 5 μL/i.c.v. bilaterally during surgery (Grieb, 2016[21]). 

### Exercise protocol 

Following obtaining the experimental and control models, the exercise protocol was performed (Khajehnasiri et al., 2018[28]). To warm up the animals, treadmill speed was initially set at 5 m/min and gradually increased to 20- 22 m/min. At the end of the 60-minute exercise, the speed was gradually reduced to 5 m/min to cool down the animals. The training was performed for 60 continuous days by treadmill training (60 min/day; 22 m/min). The mild electrical shock was rarely used to motivate animals to run. The animals on the treadmill did not exercise, but they were placed on a non-moving treadmill for 60 min per day.

### Experimental design

#### Behavioral test

Based on previous studies, the MWM test was used to evaluate spatial memory (Sadeghian et al., 2012[43]). In short, a circular black pool (155 cm in diameter and 60 cm in height) was filled with water (22 ± 1 °C) to 25 cm in depth. The pool was split into four quadrants. In the center of the northern quadrant (target quadrant) a black hidden escape platform was submerged 1 cm below the water level. The rats were placed in one of the four randomly selected quadrants, and the time spent to escape from water onto a hidden platform (escape latency) was measured. Animals were given a series of four daily trials (60 s) at the same time (10:00–12:00) for five consecutive days. When an animal could not find the platform in 60 s, it was placed slowly on the platform by the researcher and allowed to remain there for 30 s. Rats were given a 30-second rest on a platform between each trail. A video tracking system (Panasonic, Japan) connected to a computer was mounted directly above the water maze pool and recorded escape time, distance traveled, and swimming speed (Azizi-Malekabadi et al., 2012[4]; Sadeghian et al., 2012[43]).

#### Biochemical parameters

After the last day of the MWM test, all rats were anesthetized by i.p. injection of ketamine (100 mg/kg) and xylazine (10 mg/kg) (Khajehnasiri et al., 2019[29]). Then, the head was separated with a guillotine, and the hippocampus region was separated and washed with saline. The isolated hippocampus was immediately frozen in liquid nitrogen. All samples were kept in a freezer at -80 °C. At the end of the experiments, hippocampal tissue was homogenized using a mechanical homogenizer and transferred to microtubules and centrifuged at 4,000 rpm for 3 min at 3,000 rpm. Total protein in supernatants was measured by the Bradford method using bovine serum albumin as standard. The biochemical parameters and levels of biomarkers of oxidative stress were determined by the estimating total antioxidant capacity using ferric ion reducing antioxidant power (FRAP) assay and superoxide dismutase (SOD) and catalase (CAT) activities. Also, the assessment of lipid peroxidation was done by measuring malondialdehyde (MDA).

### Antioxidant enzyme activity assay

#### The measurement of the total antioxidant activity

The FRAP test for the evaluation of total antioxidant activity was performed according to the technique developed by Benzie and Strain. In brief, 1.5 ml of working FRAP reagent (25 ml 0.3 M sodium acetate buffer, 2.5 ml 0.01 MTPTZ (2, 4, 6-tri (2-pyridyl)-1, 3, 5-triazine) in 0.04 M HCl; 2.5 ml 0.02 M FeCl_3_∙6H_2_O) was blended with 50 μl of the supernatant. Then, it was incubated at 37 °C for 5 min and the absorbance was measured spectrophotometrically at 593 nm. FeSO_4_ solutions from 0.2 to1.2 mM in 1.15 % KCl were used for calibration. The results of the FRAP assay were expressed as μmol/mg of protein (Sadeghnia et al., 2013[44]).

#### The measurement of SOD activity 

The SOD activity was determined by the mixture of 0.1 ml of phenazine methosulphate (186 μM) and 1.2 ml of sodium pyrophosphate buffer (0.052 mM, pH 7.0) that was added to 0.3 ml of the 10 % homogenate supernatant. The enzyme reaction began by adding 0.2 ml of Nicotinamide adenine dinucleotide (NADH; 780 μM) and ceased after 1 min by adding 1 ml of glacial acetic acid. The amounts of chromogen were recorded by measuring color intensity at 560 nm. One SOD unit was expressed in units per mg protein (units/mg) (Khan, 2012[30]).

#### The measurement of CAT enzyme activity

The CAT activity was determined according to the Aebi method. In this regard, 30 mM hydrogen peroxide (H_2_O_2_) was used as a substrate, and 50 mM phosphate buffer (pH =7) was used as an alternative substrate in the blank. The reaction was made by adding H_2_O_2_ and the absorption was measured at 240 nm for 3 min (Ghasemi et al., 2019[20]).

#### Lipid peroxidation assay

Lipid peroxidation in the rats’ hippocampus was measured using the thiobarbituric acid reactive substance (TBARS) assay kits (Kiazist, Iran). Briefly, lipid peroxidation products (such as MDA) were mixed by 1.0 ml of 20 % TBA at 100 °C for 80 min, and 1.0 ml of 1 % TBARS was measured by 100 µl of the supernatant and incubation solution. After cooling the solution on ice, it was centrifuged at 3,000 rpm for 20 min and the absorbance of the supernatant was read at 532 nm. TBARS concentration was calculated using the standard MDA curve and expressed as nmol/mg protein (Ghasemi et al., 2019[20]).

### Statistical analysis

Data analysis was done using SPSS version 16.0. Because of the three dependent variables (traveled distance, escape latency time, and swimming speed), and two independent variables (groups and trial days), the effects of trial days, groups, and their interactions were analyzed by the multivariate analysis of variance (MANOVA) test. Also, Tukey's post-hoc test was used to compare significant differences. Furthermore, one-way ANOVA was used for assessing biochemical parameters and the statistical significance was determined by Tukey's post-hoc test. P<0.05 was regarded as statistically significant, and the results were expressed as mean ± standard deviation.

## Results

### Behavioral experiment 

The results of MANOVA showed a significant relationship between groups [F (21,840) = 7.131, *P*<0.001], and trial days [F (12,840) = 14.038, *P*<0.001] in terms of dependent variables of traveled distance, escape latency time, and swimming speed. Also, the groups and trial days interaction showed a significant relationship regarding the dependent variables [F (84,840) = 4.89, *P*<0.05].

#### Escape latency time of rats in the Morris water maze test

The MANOVA test revealed a significant difference between groups [F (7, 280) = 20.262, *P*< 0.001], trial days [F (4, 280) = 54.704, *P*<0.001)], and interactions between groups and days (F (28, 280) = 12.03; *P*<0.05). Also, the Tukey’s post-hoc test indicated that the escape latency time significantly increased in the STZ group compared with the control (*P*<0.001) and sham (*P*<0.001) groups. This parameter in the EX +STZ (*P*<0.01) and Q80+STZ (*P*<0.01) groups was significantly lower than the Ethanol+STZ group. 

In addition, there was no significant difference between the Ethanol+STZ, STZ (*P*=0.97), and off treadmill+STZ (*P*=0.98) groups. Generally, the lowest performance (highest level of latency) was found in the STZ, off treadmill+STZ, and Ethanol+STZ groups, whereas no differences were observed between the control, sham, Q80+STZ, and EX+STZ groups. Also, a significant decrease in latency time on days 3, 4, and 5 compared with days 1 and 2 (*P*<0.001) was observed, but it was significantly different from days 3 and 4 (*P*= 0.38). 

**Figure 1:**
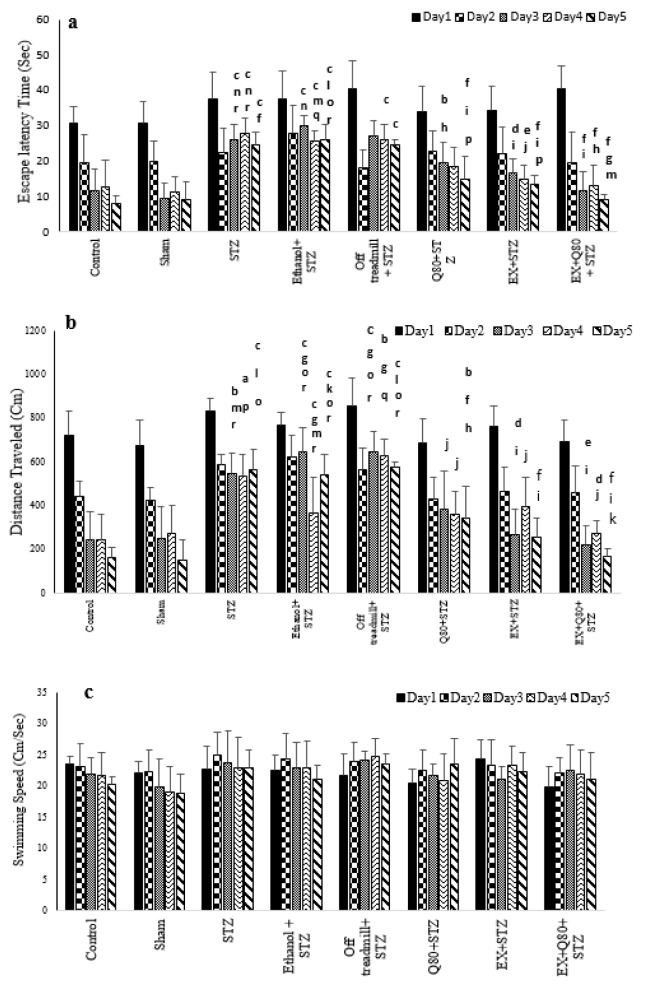
Spatial memory was evaluated as a function of training day according to the following parameters: (a) Escape latency, (b) Traveled distance, and (c) Swimming speed. Data are presented as the mean ± standard deviation (SD) (n = 7 animals). Significantly different from sham (^a^ p<0.05, ^b^ p<0.01, ^c^ P<0.001), significantly different from STZ (^d^ p<0.05, ^e^ p<0.01, ^f^ P<0.001), significantly different from Ethanol+STZ (^j^ p<0.05, ^h^ p<0.01, ^i^ P<0.001),significantly different from Q80+STZ (^g^ p<0.05, ^k^ p<0.01, ^l^ P<0.001), significantly different from EX+STZ (^m ^p<0.05, ^n^ p<0.01, ^o^ P<0.001), significantly different from Q80+EX+STZ (^p^ p<0.05, ^q^ p<0.01, ^r^ P<0.001).

Also, the results of MANOVA revealed no significant differences in the escape latency time between groups on day 1 [F (7, 48) = 1.109, *P*=0.373], and day 2 [F (7, 48) = 1.584, *P*=0.163]. Besides, the MANOVA test results indicated significant differences in the escape latency time to reach the platform [F (7,48) = 10.286, *P*<0.001] between all groups on day 3. The Tukey's post-hoc comparisons showed that the escape latency time of the swimming path on day 3 significantly increased in the STZ (*P*<0.05), off treadmill+ STZ (*P*<0.001), and Ethanol+STZ (*P*<0.001) groups compared with the sham group (Figure 1a(Fig. 1)). 

The animals of both EX+STZ (*P*<0.001) and Q80+STZ (*P*<0.05) groups showed a significantly lower latency time in comparison with the Ethanol+STZ group on day 3. Also, on day 4 [F (7,48)= 8.821, *P*<0.001] and day 5 [F (7,48)= 27.602, *P*<0.001; Figure 1a(Fig. 1)], a statistically significant change was found in the escape latency time similar to day 3. Figure 1a(Fig. 1) illustrates a remarkable reduction in latency in the EX+Q80+STZ rats than the EX +STZ (*P*<0.05), Q80+STZ (*P*<0.05), STZ (*P*<0.001), Ethanol+STZ (*P*<0.001), and off treadmill+STZ (*P*<0.001) groups on day 5. The experiment data were given in Table 1(Tab. 1).

#### Distance traveled by rats in the MWM test

The results of multivariate ANOVA showed a significant difference between the groups [F (7,280) = 24.864, *P*<0.001], and trial days [F (4,280) = 51.136, *P*< 0.001], as well as the interaction between groups and trial days [F (28,280) = 16.02, *P*< 0.05]

Tukey's post-hoc test showed that the length of the swimming path in the STZ group significantly increased than the control (*P* <0.001) and sham (*P* <0.001) groups. Also, the distance traveled in the Q80+STZ group significantly reduced compared with the STZ (*P* <0.001) group; however, EX+Q80+STZ showed a significantly shorter distance traveled than the STZ (*P* <0.001), Ethanol+STZ (*P* <0.001), and off treadmill+STZ (*P* <0.001) groups.

The average traveled distance showed a significant difference in trail days (F (4,280) = 51,136, *P* <0.001). Tukey’s post-hoc test indicated that it was significantly higher on days 1 and 2 compared with days 3 (*P* <0.001), 4 (*P* <0.001), and 5 (*P* <0.001); however, it was not significantly different than days 3 and 4 (*P*= 0. 2).

The multivariate ANOVA revealed no significant differences in distance traveled between groups on days 1 [F (7, 48) = 1.074, *P*=0.395] and 2 [F (7, 48) = 1.844, *P*=0.1]. There was a significant difference in the distance traveled to reach the hidden platform [F (7,48) = 18.592, *P*<0.001] between the groups on day 3. Tukey's post-hoc test showed that the length of the swimming path on day 3 significantly increased in the STZ (*P*<0.001), off treadmill+STZ (*P*<0.001), and Ethanol+STZ (*P*<0.001) groups compared with the sham rats (Figure 1b(Fig. 1)).

The animals in both EX+STZ (*P*<0.001) and Q80+STZ (*P*<0.01) groups showed significantly shorter distance traveled in comparison with the Ethanol+STZ group on day 3. Also, on days 4 [F (7,48)= 7.32, *P*<0.001] and 5 [F (7,48)= 34.917, *P*<0.001], a statistically significant change was found in distance traveled [F (7,48)= 34.917, *P*<0.001; Figure 1b(Fig. 1)] similar to day 3. The experiment data were given in Table 1(Tab. 1).

### The swimming speed of rats in the MWM test

The results of multivariate ANOVA showed a significant difference between the groups [F (7,280) = 2.158, *P*=0.39] and trial days [F (4,280) = 1.160, *P*=0.34] and also the interaction between groups and trial days [F (28,280) = 1.32, *P*=0.88].

The results of multivariate ANOVA regarding the swimming speed of rats to find the platform showed no significant difference between groups on days 1 [F (7, 48) = 1.146, *P*=0.351], 2 [F (7, 48) = 0.351, *P*=0.925], 3 [F (7, 48) = 0.76, *P*=0.623), 4 [F (7, 48) = 0.998, *P*=0.444], and 5 [F (7, 48) = 1.149, *P*=0.350; Figure 1c(Fig. 1)]. The experiment data were given in Table 1(Tab. 1).

### Biochemical tests

#### Measurement of the total protein

**Figure 2:**
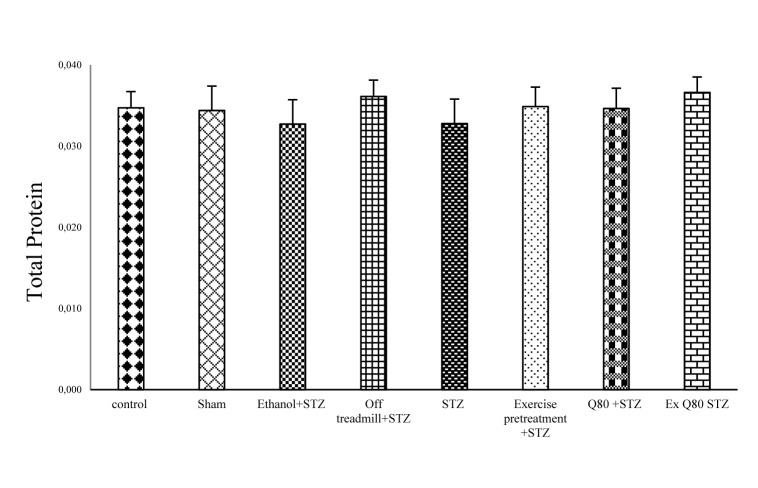
Figure 2: The effects of STZ and Q80 administration or exercise treatment on hippocampus levels of total protein. Each column and bar represent the mean ± standard deviation (SD). There was no difference between groups.

One-way ANOVA showed no significant difference in levels of total protein between the experimental and control groups [F (7, 28) = 1.965; P = 0.0797; Figure 2(Fig. 2)].

#### Effect of STZ and Quercetin on FRAP assay

**Figure 3:**
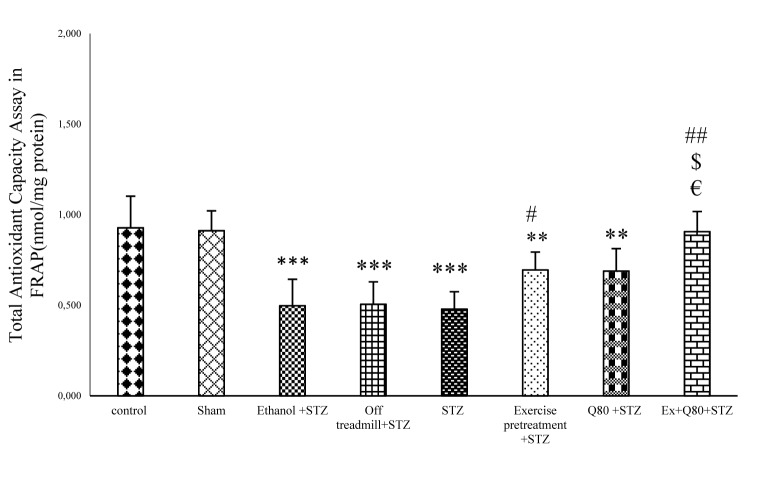
Figure 3: The effects of STZ and Q80 administration or exercise treatment on hippocampus levels of total antioxidant or Ferric Reducing Ability of Plasma (FRAP) (nmol/mg protein). Data are expressed as the mean ± standard deviation (SD). * with respect to the control group, ^# ^with respect to the STZ group, ^$^ with respect to the Exercise+STZ group, and ^€^ with respect to the Q80+STZ group.

The results of one-way ANOVA indicated a significant difference between the groups in total antioxidant activity [F (7, 28) = 110.1; P<0.001; Figure 3(Fig. 3)]. Tukey’s post-hoc test revealed that Fe^+2^ concentration in the STZ group was lower than the control (P<0.001), sham (P<0.001), and EX+STZ groups (P<0.05). Also, the EX+Q80+STZ group was higher than the STZ group (P<0.01; Figure 3(Fig. 3)). 

#### Effect of STZ and Quercetin on SOD activity

**Figure 4:**
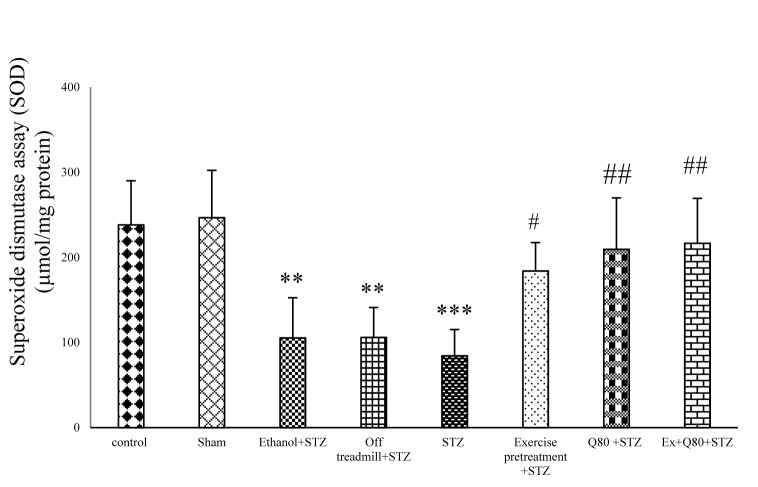
Effect of STZ and Quercetin on superoxide dismutase activity assay (SOD) (μmol/mg protein); n = 7 per group. Data are expressed as the mean ± standard deviation (SD). * with respect to the control group, and ^# ^with respect to the STZ group.

Analysis of the SOD activity in different groups showed a significant difference between groups [F (7, 28) = 11.14; P<0.001]. Tukey’s post-hoc test showed a significant difference between the EX+STZ and STZ groups (P <0.05), however, there was no significant difference between the STZ and the off treadmill+STZ groups. A significant difference was observed between the off treadmill+STZ and control groups (P<0.01). Assessment of the SOD activity revealed a significant difference between the Q80+STZ group and the STZ group (P <0.01) or Ethanol+STZ (P <0.05) group. Also, the results showed a significant difference between the Ethanol+STZ group and the control group (P <0.01). The EX+Q80+STZ group also had a significant difference with the STZ group (P <0.01), but there was no significant difference in SOD activity between the control and the EX+STZ or Q80+STZ groups (Figure 4(Fig. 4)).

#### Effect of STZ and Quercetin on CAT enzyme activity

**Figure 5:**
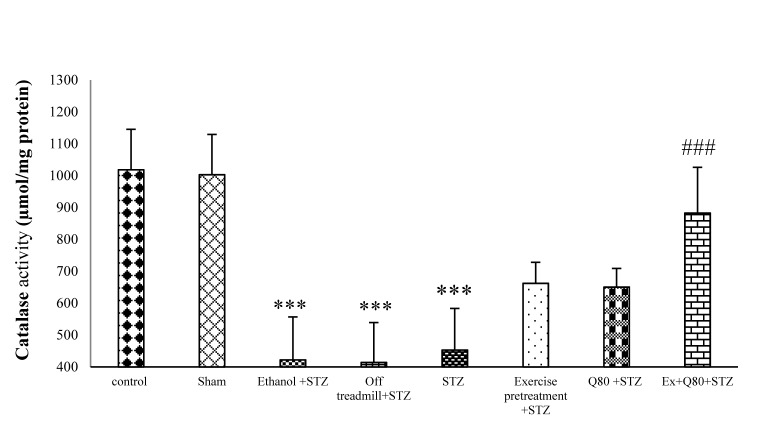
Effect of STZ and Quercetin on catalase (CAT) enzyme activity (μmol/mg protein); n = 7 per group. Data are expressed as the mean ± standard deviation (SD). * with respect to the control group, and ^#^ with respect to the STZ group.

The results of One-way ANOVA revealed significant differences between groups in CAT activity [F (7, 28) = 31.10; P <0.001]. Tukey’s post-hoc showed a significant difference between the control and STZ groups (P<0.001). The EX+Q80+STZ group was higher than the STZ group (P <0.001), but there was a significant difference between the EX+Q80+STZ group with the control (P <0.05), exercise (P <0.001) or Q80+STZ groups (P <0.001; Figure 5(Fig. 5)).

#### Effect of STZ and Quercetin on lipid peroxidation (MDA) assay

**Figure 6:**
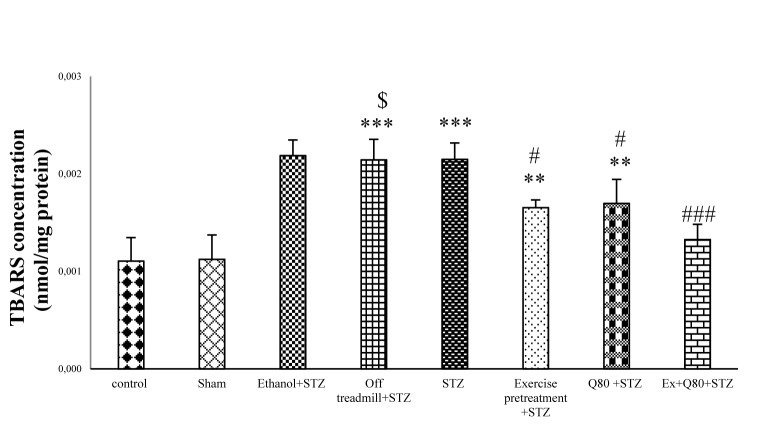
The malondialdehyde (MDA) level of hippocampus in all groups (μmol/mg protein); n = 7 per group. Data are expressed as the mean ± standard deviation (SD). * with respect to the control group,^ # ^with respect to the STZ group, ^$^ with respect to the Exercise+STZ group, and ^€ ^with respect to the Q80+ STZ group.

The results of the lipid peroxidation assay showed significant differences between groups [F (7, 48) = 19.57; P< 0.001]. According to the results, TBARS as a lipid peroxidation marker was significantly higher in STZ groups than the control (P <0.001) and EX+STZ groups (P<0.05). Also, lipid peroxidation in the Q80+STZ group was lower than the STZ group (P<0.05). The EX+Q80+STZ group also showed a significant difference with the STZ group (P<0.001), but no significant difference was found between the exercise, Q80+STZ, and control groups (Figure 6(Fig. 6)). 

## Discussion

In this study, the synergistic effect of quercetin and regular exercise on spatial memory and oxidative stress in AD rats was examined. The key results of the study were as follows: (1) Treatment with STZ showed a significant increase in escape latency and distance traveled in comparison with the control or sham groups; (2) These parameters in the EX+STZ and Quercetin 80 mg/kg+STZ groups were lower than the off treadmill+ STZ and STZ groups; (3) Cotreatment with exercise and quercetin significantly decreased escape latency and distance traveled compared with the STZ, EX+STZ, and Q80+STZ groups; (4) Fe^+2^ concentrations in the STZ group were lower than the sham and EX+STZ groups; (5) Lipid peroxidation in the STZ or off treadmill+STZ groups was more than the control and EX+STZ groups; (6) Analysis of SOD activity revealed its higher level in the EX+Q80+STZ and Q80+STZ than the STZ and off treadmill+STZ groups; (7) CAT enzyme activity in the EX+Q80+STZ group was higher than the EX+STZ and Q80+STZ groups. 

One of the most critical obtained results was that day of training caused a significant reduction in escape latency and distance traveled by comparing the rats’ performance on day 3, 4 and 5 of training. Accordingly, these data declared the improvement of spatial learning as the rats spent less time and shorter distance to find the platform. The result of escape latency was consistent with the studies indicating a decline in escape latency in the MWM test over seven days of training in the weanling mice (Barnhart et al., 2015[5]). While training, the rats recognized the platform location in the MWM; therefore, they could find it quickly without spending more time or swimming long distances.

The group receiving STZ for 21 days spent a long time and traveled longer distances than the control group. The adverse effect of STZ on spatial memory and induction of AD has been reported. The i.c.v. injection of STZ at a dose of 3 mg/kg in rats is the most appropriate method to achieve a rat model of AD (Mehla et al., 2013[36]). Recent studies have shown that STZ-induced experimental models of AD in rodents approximately mimic the age-related pathology of AD in humans (Kamat, 2015[26]; Salkovic-Petrisic et al., 2011[46]; Shingo et al., 2013[51]). Recently, the concept that AD may represent a brain-specific form of diabetes mellitus and coined the term “type 3” diabetes has been proposed. Due to similar mechanisms, it has suggested that insulin may be a promising therapeutic agent to reduce impaired cognitive functions (Grieb, 2016[21]; Shingo et al., 2013[51]). Other important features of the rat i.c.v. STZ model that resemble findings in brains from AD patients are as follows: memory deficits, progressive cholinergic abnormalities (Kamat, 2015[26]), loss of GLUT_2_ expression, glucose hypometabolism, and other pathophysiological changes, such as processing of amyloid precursor protein (APP), as well as changes in synaptic function, protein kinases, and insulin signaling (Grieb, 2016[21]).

Lee et al. (2014[32]) stated that the expression of insulin / IGF signaling-related genes is predominantly disrupted in the frontal cortex and hippocampus of monkeys injected with STZ and this is similar to the early stage of AD (Lee et al., 2014[32]). Also, mitochondrial abnormalities and tissue oxidative stress may be associated with glial activation and TNF-α and free radical production following i.c.v. injection of STZ that occur earlier than apoptosis and synaptic toxicity (Grieb, 2016[21]). Administration of the STZ in rodent brain has been shown to cause neuroinflammation, oxidative stress, and biochemical alterations, which is considered as a valid experimental model of the early pathophysiological changes in neurodegeneration (Kamat, 2015[26]). 

Also, our result showed that an i.c.v. injection of STZ disrupted the balance in the oxidant-antioxidant system, and it caused oxidative stress in the brain, however, the underlying mechanism is not clearly understood. Also, the total antioxidant activity in the STZ group decreased compared with the control group. The concentration of TBARS, as an index for lipid peroxidation (initiating oxidative stress) in the STZ group, was higher than the control group, and also antioxidant enzyme activities (SOD and CAT) significantly decreased in the STZ group than the control group. In line with our results, other studies have shown that STZ injection caused an increase in lipid peroxidation, oxidative stress (Sharma and Gupta, 2001[50]), MDA, and glutathione (Veerendra Kumar and Gupta, 2003[57]). Exercise pretreatment and STZ can counteract the harmful effects of STZ and improve both learning and memory impairment and oxidative damage in STZ rats. Based on our results, the time spent and distance traveled to find the platform in the EX+STZ group were significantly less than the STZ group. Also, in this group, total antioxidant activity significantly increased compared with the STZ group. However, the effect of exercise on oxidative damage and antioxidant levels of the brain is controversial (Cechetti et al., 2008[11]; Suzuki et al., 1983[55]). The different obtained results are possibly due to variations in exercise types, as well as different intensities and duration of exercise protocols, age, sex and race of the rats (Radak et al., 2007[40]) or affecting different regions of the brain (Cechetti et al., 2008[11]; Endo et al., 2013[15]; Radak et al., 1996[39]). Navarro et al. (2004[38]) concluded that regular moderate exercise reduced oxidative stress in middle-aged mice, however, no difference was found in older mice (Navarro et al., 2004[38]). High-intensity exercise causes mitochondrial dysfunction, increases TBARS levels in the frontal cortex in mice (Aguiar et al., 2008[1]) and also reduces the brain's antioxidant levels (Tsakiris et al., 2006[56]). Also, after two weeks of moderate-intensity exercise, no significant change was observed in oxidative stress parameters in the hippocampus of the rats (Cechetti et al., 2008[11]). Our results suggested that treatment with quercetin could significantly improve the adverse effects of STZ on learning and memory. In terms of time spent to find the platform and distance traveled, the Q80+STZ group spent a shorter time than the STZ group. The effectiveness of quercetin, such as its neuroprotective effects on cancer and spatial memory performance has been reported. In the present study, long-term treatment with quercetin induced neuroprotective effects in the rat’s model of AD. Previous reports have shown that treatment with quercetin (5-20 mg/kg orally, twice daily for 30 days) in diabetic rats prevented the cognitive dysfunction in the MWM and elevated plus maze tests (Bhutada et al., 2010[7]). Besides, Mohammadi et al. showed that quercetin at a dose of 50 mg/kg prevents oxidative stress parameters in cases with stress stimulation and impairment of learning and memory (Mohammadi et al., 2014[37]). Also, quercetin can inhibit the progression of lipid peroxidation and enhance the ability of the antioxidant defense system in the human body (Schmitt-Schillig et al., 2005[47]). The results also showed that the total antioxidant activity increased in the Q80+STZ group compared with the STZ group. Lipid peroxidation also significantly decreased in the Q80+STZ group than the STZ group. The body's antioxidant defense systems, including SOD, CAT, and glutathione peroxidase prevent oxidative processes and protect the body against oxidative damage (Gutteridge, 1994[22]). SOD is the body's main antioxidant enzyme for the elimination of free radicals and its content and activity can reflect the body's ability to remove free radicals. MDA is one of the products of lipid peroxidation induced by free radicals in the body (Liu et al., 2006[33]). Previously, the benefits of interventions, such as exercise or antioxidant supplements alone on oxidative stress or improving brain function in humans and animals have been studied (Freitas et al., 2017[18]; Kawamura and Muraoka, 2018[27]). However, few studies have been conducted on the cotreatment effect of exercise and antioxidant supplements on oxidative stress in the different areas of the body. It was predicted that the combination of these two factors can maximize their performances and lead to their synergistic effects (Daneshvar et al., 2013[14]), however, some studies have shown that the injection of quercetin did not affect plasma cytokine before, during, or after exercise (Chou, 2012[13]) and also some investigations have revealed the results in contrast to this theory (Casuso et al., 2014[10]; Ruiz et al., 2015[42]). Nevertheless, some studies have shown the synergic effects of these factors. To date, the nature of the interplay between antioxidant and exercise has not been well understood (Bowtell and Kelly, 2019[8]). Based on the results, the combination of regular exercise and quercetin was more effective than using these factors alone. According to the results of the spatial memory test, the distance traveled to find the platform in the EX+Q80+STZ group was significantly reduced compared with the STZ, Q80+STZ, and EX groups. Exercise alone, like quercetin, had the same effect on the antioxidant system, but the combination of these two factors enhanced antioxidant performance. Quercetin donates hydroxyl hydrogen to free radicals and leads to stabilitation and prevents oxidative damage. In addition, moderate exercise increases antioxidant defense (Vincent et al., 2007[58]). The single and combined effects of exercise and antioxidant supplementation on brain functions in young and old mice showed that antioxidant supplementation alone worsened cognitive activity during aging. Exercise eliminated disorders associated with aging and improved learning in young mice. The combination of exercise and antioxidants improved cognition (Sidhu, 2015[52]). Song et al. (2012[54]) also found that regular exercise and acetyl L-carnitine alone and a combination significantly reduced oxidative stress and increased neurogenesis in the hippocampus, but no improvement in cognitive function was observed (Song et al., 2012[54]). The differences in the results of these studies and the present study could be due to differences in the type and intensity of exercise and the used antioxidants.

## Conclusions

According to our results, the injection of STZ impaired spatial memory and increased oxidative stress in the hippocampus. It seems that the combined exercise pretreatment and quercetin induced the synergistic effects and significantly improved the spatial memory impairment and oxidative stress induced by STZ injection. This effect was more desirable than their single-use and was able to recover spatial memory and restore the antioxidant status to the normal levels.

### Limitation and future directions

The present study had some limitations. First, in the current research, only one-month moderate exercise and the effect of a single dose of quercetin were investigated. Future studies are recommended to consider different conditions of intensity and duration of exercise, as well as different doses of quercetin. Second, AD was defined as the formation of amyloid-beta plaques, and also evidence has proposed that oxidative stress may enhance the production and accumulation of amyloid-beta. In the present study, the effects of exercise and quercetin on oxidative stress factors and their roles in spatial memory were investigated; however, AD brain histological examination was not performed to evaluate the amyloid-beta plaques alteration. Third, this study determined the effectiveness of intraperitoneal injection of quercetin to change oxidative stress factors, in the 3-4-month rat's hippocampus. The effect of pretreatment with quercetin by oral administration (the routine method for drug administration) and regular moderate exercise in the aged rats model of AD should be investigated in future studies and also the changes in stress oxidative factors and stress-activated protein kinase (SAPK) pathways that are activated by oxidative stress are recommended to be considered.

## Acknowledgements

The authors would like to express their gratitude to the staff of the University of Tabriz for helping us to carry out this project.

## Disclosure of potential conflicts of interest

The authors declare that they have no conflict of interest. 

## Figures and Tables

**Table 1:**
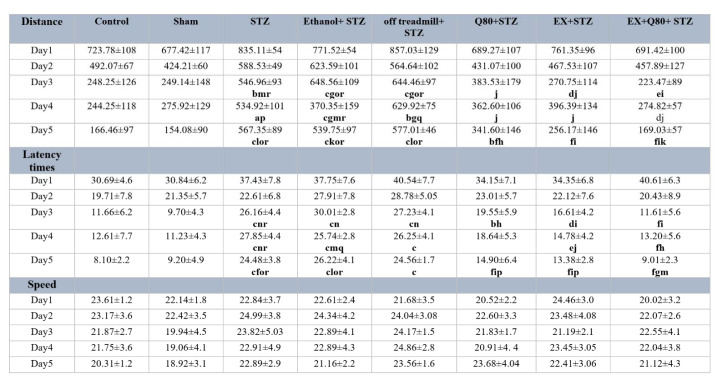
The data for the escape latency, traveled distance, and swimming speed were shown. Data are presented as the mean + standard deviation (SD) (n = 7 animals). Significantly different from sham (^a^ p<0.05, ^b^ p<0.01, ^c^ P<0.001), significantly different from STZ (^d^ p<0.05, ^e^ p<0.01, ^f^ P<0.001), significantly different from Ethanol+STZ (^j^ p<0.05, ^h^ p<0.01, ^i^ P<0.001), significantly different from Q80+STZ (^g^ p<0.05, ^k^ p<0.01, ^l^ P<0.001), significantly different from EX+STZ (^m^ p<0.05, ^n^ p<0.01, ^o^ P<0.001), significantly different from Q80+EX+STZ (^p^ p<0.05, ^q^ p<0.01, ^r^ P<0.001).
